# Elevated expression of CDK4 in lung cancer

**DOI:** 10.1186/1479-5876-9-38

**Published:** 2011-04-11

**Authors:** Aibing Wu, Bin Wu, Jinsong Guo, Weiren Luo, Dong Wu, Huiling Yang, Yan Zhen, Xiaoli Yu, Hao Wang, Ying Zhou, Zhen Liu, Weiyi Fang, Zhixiong Yang

**Affiliations:** 1 Cancer Research Institute of Southern Medical University, 510515, Guangzhou, PR China; 2 Department of Respiratory Medicine, Affiliated Hospital of Guangdong Medical College, 524000, Zhanjiang, PR China; 3 Department of Pathology, Medical College of Guangzhou, 510450, Guangzhou, PR China; 4 School of Pharmacy, Guangdong Medical College, 523808, Dongguan, PR China; 5 Cancer Center, Affiliated Hospital of Guangdong Medical College, 524000, Zhanjiang, PR China; 6 Department of Bioinformatics, Southern Medical University, 510515, Guangzhou, PR China

## Abstract

**Background**

The aim of the present study was to analyze the expression of Cyclin-dependent kinase 4 (*CDK4*) in lung cancer and its correlation with clinicopathologic features. Furthermore, the involvement of *CDK4*-mediated cell cycle progression and its molecular basis were investigated in the pathogenesis of lung cancer.

**Methods**

Using immunohistochemistry analysis, we analyzed *CDK4 *protein expression in 89 clinicopathologically characterized lung cancer patients (59 males and 30 females) with ages ranging from 36 to 78 years and compared them to 23 normal lung tissues. Cases with cytoplasmic and nuclear *CDK4 *immunostaining score values greater than or equal to 7 were regarded as high expression while scores less than 7 were considered low expression. The correlation between the expression level of *CDK4 *and clinical features was analyzed. Furthermore, we used lentiviral-mediated shRNA to suppress the expression of CDK4 and investigate its function and molecular mechanism for mediating cell cycle progression.

**Results**

The expression level of *CDK4 *protein was significantly increased in lung cancer tissues compared to normal tissues (*P *< 0.001). In addition, high levels of *CDK4 *protein were positively correlated with the status of pathology classification (*P *= 0.047), lymph node metastasis (*P *= 0.007), and clinical stage (*P *= 0.004) of lung cancer patients. Patients with higher *CDK4 *expression had a markedly shorter overall survival time than patients with low *CDK4 *expression. Multivariate analysis suggested the level of *CDK4 *expression was an independent prognostic indicator (*P *< 0.001) for the survival of patients with lung cancer. Use of lentiviral-mediated shRNA to inhibit the expression of *CDK4 *in lung cancer cell line A549 not only inhibited cell cycle progression, but also dramatically suppressed cell proliferation, colony formation, and migration. Furthermore, suppressing *CDK4 *expression also significantly elevated the expression of cell cycle regulator *p21*

**Conclusion**

Overexpressed *CDK4 *is a potential unfavorable prognostic factor and mediates cell cycle progression by regulating the expression of *p21 *in lung cancer

## Background

Lung cancer is the world's most prevalent cancer according to the World Health Organization, with 1.2 million new cases every year. Nearly all lung cancers arise due to smoking and men are more frequently diagnosed than women. However, a rise in female smoking worldwide has started reversing the trend.

In China, about 300,000 lung cancer patients (23/100,000) are diagnosed each year[1]. Unfortunately, most lung cancer patients tend to present with an advanced stage of disease due to its deep location within the lungs and lack of symptoms during early stages. This may contribute to the overall poor prognosis of most lung cancer patients. Therefore, it is of great interest to identify factors which provide early diagnosis, more accurate prognosis prediction, and allow development of novel therapeutic strategies.

Genetic abnormalities found in lung cancer typically affect two general classes of genes: oncogenes and tumor suppressors. Cancer-promoting oncogenes are typically activated in cancer cells, giving those cells new properties, such as hyperactive growth and division, protection against programmed cell death, or loss of respect for normal tissue boundaries. *CDK4 *is part of the cyclin-dependent kinase family. The protein encoded by this gene is a member of the Ser/Thr protein kinase family and is highly similar to the gene products of S. *cerevisiae *cdc28 and S. *pombe *cdc2. It is a catalytic subunit of the protein kinase complex important for G1 cell cycle progression. Transition through G1-S phases, is controlled by the regulatory subunits D-type cyclins(*CDK4 *and *CDK6*) and *CDK *inhibitor *p16*(*INK4a*). Marval *et al. *found that *CDK4 *has higher oncogenic activity than cyclin D1(*CCND1*) and it markedly enhanced malignant skin tumorigenesis in *CDK4 *transgenic mice[2]. Furthermore, overexpression of *CDK4 *has been showed in many tumor types, including oral squamous cell carcinoma[3], pancreatic endocrine tumors[4], lung cancer[5,6], and nasopharyngeal carcinoma[7], suggesting that *CDK4 *is a key factor in promoting the initiation and development of tumors.

In order to clarify the role of *CDK4 *in the pathogenesis of lung cancer, we explored the correlation of its protein expression with clinicopathologic features of lung cancer patients. We found that the expression levels of *CDK4 *were higher in lung cancer tumors compared to those in normal lung tissues. This increased *CDK4 *expression was associated with the progression and poor prognosis of lung cancer patients. Furthermore, suppressing the expression of *CDK4 *elevated tumor suppressor *p21 *expression, which may function to reduce cell proliferation and migration.

## Materials and methods

### Sample collection

Eighty-nine (89) paraffin-embedded lung cancer and 23 normal lung samples were obtained from the First Affiliated Hospital of Guangdong Medical School, Zhanjiang City, China. In the 89 lung cancer cases, there were 59 males and 30 females with ages ranging from 36 to 78 years. The clinical follow-up time of patients ranged from 6 to 55 months. For use of these clinical materials for research purposes, prior consent from the patients and approval from the Ethics Committees of this hospital was obtained. Histological classification and clinicopathologic staging of the samples were performed according to the rules of according to the WHO histologic classification.

### Immunohistochemistry

Paraffin sections (4 μm) from samples were deparaffinized in 100% xylene and re-hydrated in descending ethanol series and water according to standard protocols. Heat-induced antigen retrieval was performed in 10 mM citrate buffer for 2 min at 100°C. Endogenous peroxidase activity and non-specific antigen were blocked with peroxidase blocking reagent containing 3% hydrogen peroxide and serum, followed by incubation with goat anti-human *CDK4 *antibody (1:100) (Santa, MA, USA) for overnight at 4°C. After washing, the sections were incubated with biotin-labeled rabbit anti-goat antibody for 10 min at room temperature, and subsequently were incubated with streptavidin-conjugated horseradish peroxidase (HRP) (Maixin Inc, China). The peroxidase reaction was developed using 3, 3-diaminobenzidine chromogen solution in DAB buffer substrate. Sections were visualized with DAB and counterstained with hematoxylin, mounted in neutral gum, and analyzed using a bright field microscope.

### Evaluation of staining

The immunohistochemically stained tissue sections were reviewed and scored separately by two pathologists blinded to the clinical parameters. Expression of *CDK4 *in the nucleus and in the cytoplasm was independently evaluated. For cytoplasmic staining, the score was evaluated according to the sum of cytoplasm staining intensity and the percentage of positive staining areas of cells. The staining intensity was scored as previously described(0-3) [8,9] and the percentage of positive staining areas of cells was defined as a scale of 0 to 3 where 0 represents <10%, 1 is 10-25%, 2 is 26-75%, and 3 is ≥76%. For nuclear staining, the staining score was defined based on the sum of nuclear staining intensity and the number of positive nuclear staining. Nuclear staining intensity score was consistent with cytoplasm and positive nuclear staining scores were defined as follows: 0 represents <10%, 1 is 10-50%, 2 is 51-80%, and 3 is ≥80%. The sum of the cytoplasm and nuclear staining scores was used as the final staining score for *CDK4 *(0-12). For statistical analysis, a final staining score of 0-6 or 7-12 was respectively considered to be low or high expression.

### Establishment of lung cancer A549 cell line with stably expressing shRNA-*CDK4*

We selected two sequences(CDK4 509: Sense:5' CGCGTCCCCGCATGTAGACCAGGACCTAAGTTCAAGAGACTTAGGTCCTGGTCTACATGCTTTTTGGAAAT 3' Antisense:5'CGATTTCCAAAAAGCATGTAGACCAGGACCTAAGTCTCTTGAACTTAGGTCCTGGTCTACATGCGGGGA 3') CDK4 1097 Sense:5'CGCGTCCCCGCAGCACTCTTATCTACATAATTCAAGAGATTATGTAGATAAGAGTGCTGCTTTTTGGAAAT 3'; Antisense:5'CGATTTCCAAAAAGCAGCACTCTTATCTACATAATCTCTTGAATTATGTAGATAAGAGTGCTGCGGGGA 3') for targeting the *CDK4 *gene using the BLOCK-It RNAi Designer (Invitrogen, Carlsbad, CA). The preparation of lentiviral vectors expressing human *CDK4 *short hairpin RNA (shRNA) was performed using the pLVTHM-GFP Lentiviral RNAi Expression System. Replication-incompetent lentivirus was produced by cotransfection of the pLVTHM/*CDK4-*shRNA expression vector and ViraPower packaging mix containing an optimized mixture of two packaging plasmids: psPAX2 and pMD2.G into 293FT cells. Lung cancer A549 cells were infected with lentiviral particles containing specific or negative control vectors and the single colony with strong GFP expression was selected to establish stable silencing cell lines. The total RNA of these cell clones was isolated, and the levels of *CDK4 *mRNA were measured using real-time PCR examination.

### Western blot Analysis

Cells were lysed in RIPA Buffer (50 mM Tris-HCl pH 8.0, 1 mM EDTA pH 8.0, 5 mM DTT, 2% SDS), and protein concentration was determined using BCA assay (Beyotime Inc, China). Total protein (30 μg) was resolved using a 10% SDS-PAGE gel and electro-transferred to polyvinylidene fluoride membranes (Invitrogen, Carlsbad, CA), and blocked with 5% nonfat dry milk in Tris-buffered saline, pH 7.5. Membranes were immunoblotted overnight at 4°C with rabbit polyclonal anti-*CDK4 *antibody(1:500), anti-*ACTB *antibody(1:400) and *p21*(1:200)(Santa Cruz Biotechnology, CA, USA). An HRP-conjugated anti-rabbit IgG antibody was used as the secondary antibody (Zhongshan Inc, China).

### Cell Proliferation

Cell proliferation was analyzed using MTT assay (Sigma, St. Louis, USA). Briefly, 1 × 10^3 ^cells were seeded into a 96-well plate with quadruplicate repeat for each condition. After 24 h of incubation, MTT reagent was added to each well and incubated for 4 h. The formazan crystals formed by viable cells were then solubilized in DMSO and measured at 490 nm for the absorbance (A) values. Each experiment was performed in triplicate.

### Colony Formation Assay

About 100 cells were added to each well of a 6-well culture plate, and each cell group contained 2 wells. After 2 weeks of incubation, cells were washed twice with PBS and stained with Giemsa solution. The number of colonies containing ≥ 50 cells was counted under a microscope. The colony formation efficiency was calculated as: efficiency = (number of colonies/number of cells inoculated) × 100%. Each experiment was performed in triplicates.

### Cell Cycle

To evaluate cell cycle distribution, cells were seeded on 10 cm-diameter plates in RPMI 1640 culture medium containing 10% NBCS. After 48 h of incubation, a total of 1 × 10^6 ^cells were harvested, rinsed with cold PBS, and fixed with 70% ice-cold ethanol for 48 h at 4°C. Fixed cells were rinsed with cold PBS followed by incubation with PBS containing 10 μg/mL propidium iodide and 0.5 mg/mL RNase A for 15 min at 37°C. The DNA content of labeled cells was acquired using FACS Caliber cytometry (BD Biosciences). Each experiment was performed in triplicates.

### *In Vitro *Cell Migration Assay

Cells growing in the log phase were treated with trypsin and re-suspended as single-cell solution. A total of 1 × 10^5 ^cells were seeded on a fibronectin-coated polycarbonate membrane insert in a transwell apparatus (Corning Inc., Corning, NY). In the lower chamber, 600 μl of RPMI 1640 with 10% NBCS was added as chemoattractant. After the cells were incubated for 12 h, the insert was washed with PBS, and cells on the top surface of the insert were removed by a cotton swab. Cells adhering to the lower surface were fixed with methanol, stained with Giemsa, and counted under a microscope in five predetermined fields (× 200). All assays were independently repeated at least three times.

### Expression examination of Cell cycle factors

Changes in expression of cell cycle regulators *CDK1, CDK2, CDK6, CCND1, p15, p16, p21*, and *p27 *were first detected by real-time PCR in pLVTHM/*CDK4-*shRNA and control expression vector. Subsequently, genes with markedly differential expression were further validated by western blot. Real-time PCR and western blot were carried out as described above.

### Statistical analysis

All data were analyzed for statistical significance using SPSS 13.0 software. The Mann-Whitney U test was applied to the examination of relationship between *CDK4 *expression levels and clinicopathologic characteristics. Survival analysis was performed using Kaplan-Meier method. Multivariate Cox proportional hazards method was used for analyzing the relationship between the variables and patient's survival time. One-way ANOVA was used to determine the differences between groups for all *in vitro *analyses. A *P *value of less than 0.05 was considered statistically significant.

## Results

### Immunohistochemical analysis of *CDK4 *protein expression in lung cancer and normal lung tissues

**Figure 1:**
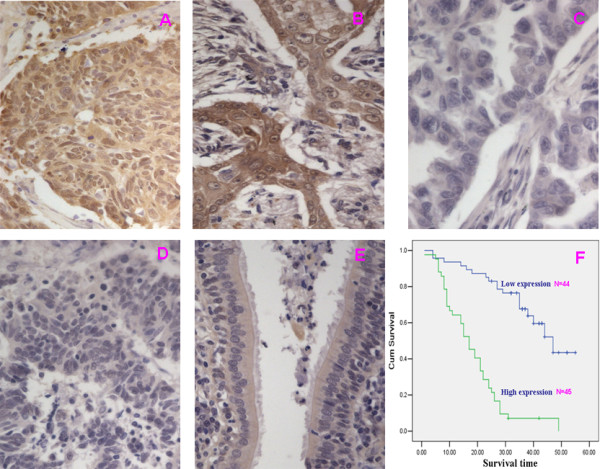
**Expression of *CDK4 *protein predicts lung cancer patients' survival time**. **A and B**: Strong expression of *CDK4 *in lung cancer samples; **C and D**: Weak expression of *CDK4 *in lung cancer sample; **E**:Weak expression of CDK4 in normal lung tissue. **F**. Kaplan-Meier survival analysis of overall survival duration in 89 lung cancer patients according to *CDK4 *protein expression. The log-rank test was used to calculate p values.

We measured the expression levels and subcellular localization of *CDK4 *protein in 89 archived paraffin-embedded lung cancer samples and 23 normal lung tissues using immunohistochemical staining (Figure 1A-E). Specific *CDK4 *protein staining was found in the cytoplasm and nucleus of normal and malignant lung tissues. Furthermore, we observed that in 50.6% (45/89) of lung cancer samples, *CDK4 *protein was highly expressed. In comparison, only 8.7%(2/23) of normal lung samples had highly expressed *CDK4 *protein, significantly lower than that in the lung cancer samples (*P *< 0.001) (Table 1).

**Table 1:** Protein expression of CDK4 between lung cancer and normal lung tissues

Group		Protein expression		P value
	**Cases**	**High expression**	**Low expression**	
Cancer	89	45(50.6%)	44 (49.4%)	
Normal	23	21(8.7%)	2 (91.3%)	0.000

### Relationship between clinicopathologic characteristics and *CDK4 *expression in lung cancer patients

The relationship between clinicopathologic characteristics and *CDK4 *expression levels in individuals with lung cancer are summarized in Table 2. We did not find a significant association of *CDK4 *expression levels with patient's age, sex, smoking, degree of differentiation, tumor size (T classification), or status of distant metastases (M classification) in 89 lung cases. However, we observed that the expression level of *CDK4 *was positively correlated with the status of pathology classification(*P *= 0.047) lymph node metastasis (N classification) (N0-N1 *vs*. N2-N3) (*P *= 0.007), and clinical stage (I-II *vs. *III-IV) (*P *= 0.004) in lung cancer patients (Table 2).

**Table 2:** Correlation between the clinicopathologic characteristics and expression of CDK4 protein in lung cancer

		CDK4 (%)		
			
Characteristics	n	High expression	Low expression	*P*
Gender				
Male	59	30(50.8%)	29 (49.2%)	
Female	30	15(50%)	15 (50%)	1.000
Age(y)				
≥65	39	21 (53.8%)	18 (46.2%)	
<65	50	24 (48%)	26(52%)	0.671
Smoking				
Yes	38	23 (60.5%)	15 (39.5%)	
No	51	22 (43.1)	29 (56.9)	0.135
Pathology classification				
squamous cell carcinoma	39	15(38.5%)	24(61.5%)	
adenocarcinoma	46	17(40%)	29(60%)	
small cell undifferentiated carcinoma	4	3(75%)	1(25%)	0.047*
Differentiated degree				
High	25	9(36%)	16(64%)	
middle	34	21(61.8%)	13(38.2%)	
Low or undifferentiated	30	15(50%)	15(50%)	0.150*
T classification				
T1+T2	71	32(45.1%)	39(54.9%)	
T3+T4	18	13(72.2%)	5(27.8%)	0.063
N classification				
N0+N1	58	23 (39.7%)	35 (60.3%)	
N2+N3	31	22 (71%)	9 (29%)	**0.007**
Distant metastasis				
Negative	3	3 (100%)	0 (0%)	
Positive	86	42 (48.8%)	44 (51.2%)	0.242
Clinical stage				
I～II	55	21(38.2%)	34(61.8%)	
III～IV	34	24 (70.6%)	10(29.4%)	**0.004**

*Kruskal Wallis Test

### Survival analysis

To investigate the prognostic value of *CDK4 *expression for lung cancer, we assessed the association between the expression levels and patient survival using Kaplan-Meier analysis with the log-rank test. In 89 lung cancer cases with prognosis information, we observed that the level of *CDK4 *protein expression was significantly correlated with the overall survival of lung cancer patients (Figure 1F). Patients with higher levels of *CDK4 *expression had poorer survival rates than those with lower levels of *CDK4 *expression (*P *< 0.001). In addition, smoking, degree of tumor differentiation, T/N/M classifications and clinical stages were also significantly correlated with patients' survival (*P *= 0.05, *P *= 0.004, *P *= 0.018, *P *= 0.003, *P *= 0.039, and *P *< 0.001 respectively). To determine whether *CDK4 *is an independent prognostic factor for lung cancer, we performed multivariate analysis of *CDK4 *expression adjusted for the same parameters. The results indicated that the level of *CDK4 *expression was an independent prognostic factor for lung cancer (*P *< 0.001) (Table 3).

**Table 3:** Summary of univariate and multivariate Cox regression analysis of overall survival duration

	Univariate analysis			Multivariate analysis		
		
Parameter	*P*	HR	95%CI	*P*	HR	95%CI
Age						
≥65vs. <65 years	0.573	1.160	0.692-1.946			
Gender						
Male vs. female	0.061	0.574	0.322-1.025			
Smoking						
Yes vs. No	0.05	0.586	0.344-0.999	0.145	0.656	0.372-1.156
Pathology classification						
Squamous vs. Adenocarcinoma vs. Small cell undifferentiated	0.883	1.036	0.648-1.656			
Differentiation degree						
High vs. Middle vs.Low	0.004	1.660	1.176-2.343	**0.001**	2.076	1.370-3.144
T classification						
T_1_-T_2 _vs. T_3_-T_4_	0.018	2.020	1.130-3.612	0.609	0.819	0.381-1.759
N classification						
N_0_-N1 vs. N_2--_N_3_	0.003	2.259	1.323-3.860	0.996	1.003	0.273-3.692
M classification						
M_0 _vs. M_1_	0.039	3.436	1.066-11.078	0.088	3.666	0.825-16.293
Clinical stage						
Ⅰ-Ⅱ vs. Ⅲ-Ⅳ	0.000	2.586	1.515-4.412	0.470	1.605	0.445-5.787
CDK4 expression						
High vs. Low *	0.000	6.420	3.473-11.867	**0.000**	6.714	3.329-13.451

### Reduced *CDK4 *Expression Suppressed the Proliferation of lung cancer cells *in vitro*

**Figure 2:**
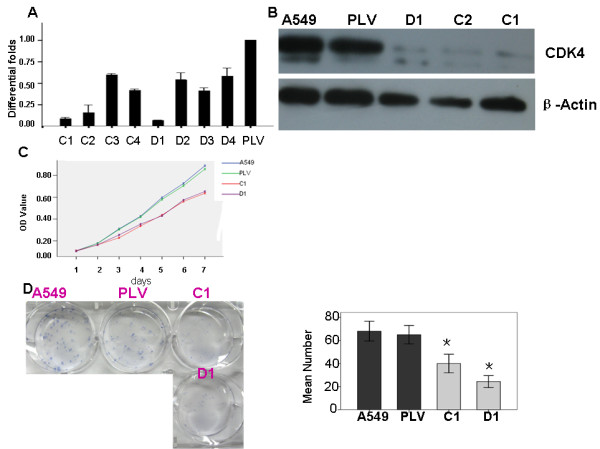
**Down-regulation of CDK4 inhibited cell growth in vitro**. **A**. Markedly reduced mRNA expression of CDK4 after shRNA-*CDK4*: 8 single clone cells(C1-C4,D1-D4) compared with PLV-Ctr by real-time PCR. **B**. Significantly decreased protein expression of *CDK4 *was found in shRNA-CDK4 cells(C1,C2,D2) compared with PLV-Ctr and A549 cells by western blot. ACTB was used as internal control. **C**. The cell growth of parental A549 cells and their stable derivatives, PLV-Ctr and shRNA-*CDK4*, was examined by MTT assay over a seven-day period. *P < 0.05, as compared to A549 and PLV-Ctr cells. **D**. The anchorage-dependent growth of parental A549 cells and their stable derivatives, PLV-Ctr and shRNA-*CDK4*, was examined by plate colony formation assay. *P < 0.05, as compared to A549 and PLV-Ctr cells.

To study the biological function of *CDK4*, we used a lentiviral vector containing shRNA to specifically target and stably knock down the expression of *CDK4 *in A549 cells, a lung cancer cell line with high endogenous levels. Eight stably transfected cell clones were obtained (C1, C2, C3, C4, D1, D2, D3, D4) (Figure 2A). Real-time PCR analysis showed that *CDK4 *mRNA expression in C1, C2, and D1 cells was markedly reduced compared to empty vector control clone A549 cells(PLV-Ctr). Further, decreased expression of *CDK4 *protein was confirmed by western blotting in these three clones compared to PLV-Ctr and A549 cells(Figure 2B). C1 and D1 clones with significantly reduced *CDK4 *protein expression were finally chosen for further experiments.

We examined the effect of decreased *CDK4 *expression on lung cancer cell growth in vivo. Using an MTT assay, we found that the parental lung cancer A549 cells had a similar growth rate as PLV-Ctr cells over a seven-day period, the growth of shRNA-*CDK4 *cells was significantly slower than the former two lines from day 3 (P < 0.05) (Figure 2C). Interestingly, this result was also consistent in the plate clone formation test. Both the parental A549 cells and the PLV-Ctr cells formed a similar number of colonies on plate over a two-week period [(68 ± 8.54) vs. (65 ± 8.00)]. In contrast, knocking down endogenous *CDK4 *could dramatically reduce the number of colonies in C1 cells(40 ± 8.0) and D1 cells(24.33 ± 5.13) (P < 0.05) (Figure 2D).

### Knock-down of *CDK4 *Inhibited Migration and Cell Cycle Progression

**Figure 3:**
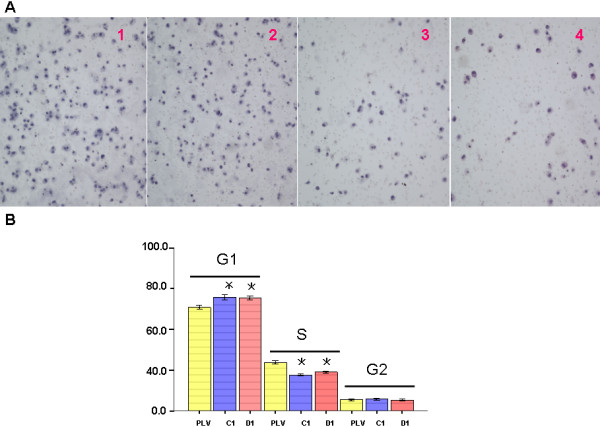
**Reduced *CDK4 *expression inhibited cell migration and cell cycle progression *in vitro***. **A**: The migrating capability of parental A549 cells and their stable derivatives, PLV-Ctr and shRNA-*CDK4*, was examined by transwell and boyden chamber assay. **B**: Cell cycle profile was determined by FACS Caliber cytometry. Data were presented as mean ± SD for three independent experiments. *P < 0.05, as compared to PLV-Ctr and A549 cells.

Cell migration is a key step during tumor development and metastasis. We tested the ability of A549 cells to migrate through the 8 μm pores on the polycarbonate membrane, and found that the knock-down of endogenous *CDK4 *expression could significantly decrease cell migration of C1 cells(114 ± 26.75) and D1 cells(80 ± 7.31) compared to the parental cells(288.2 ± 41.78) or PLV-Ctr cells (254 ± 34.28) (P < 0.05) (Figures 3A).

We measured the alteration of cell cycle progression after *CDK4 *knock-down. Using flow cytometry analysis, we found that *CDK4*-deficient cells showed a significant increase in G1 phase population cells and a decrease in S phase cells compared to the PLV-Ctr and the parental A549 cells (P < 0.05) (Figure 3B).

### *CDK4 *Inhibited the Expression of p21 in A549 cells

**Figure 4:**
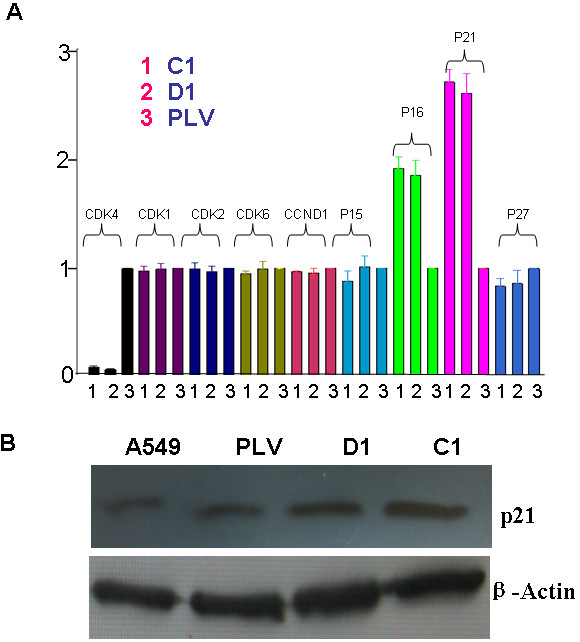
**Down-regulation of *CDK4 *elevated the expression of *p21 *protein**. **A**.mRNA expression of *p21 *was inhibited in shRNA-*CDK4 *cells compared to PLV-Ctr cells and parental A549 cells. **B**: *p21 *protein expression was suppressed in shRNA-*CDK4 *cells compared to PLV-Ctr cells and parental A549 cells. Data were presented as mean ± SD for three independent experiments. *P < 0.05.

The above results indicated that over-expression *CDK4 *may play an important role in promoting the development of lung cancer. We further examined the effect of *CDK4 *on the expression of key regulators of G1-S cell cycle transition including *CDK1, CDK2, CDK6, CCND1, p15, p16, p21*, and *p27*. Real-time PCR indicated that reducing the levels of *CDK4 *significantly activates the expression of tumor suppressor *p21 *by 3.12-fold(Figure 4A). Further, we measured the protein levels of *p21 *in cells deficient of *CDK4 *by western blot. *CDK4*-deficient cells had increased levels of *p21 *protein compared to the parental A549 cells and cells expressing the control vector (Figure 4B). Our results suggest that *CDK4 *may be involved in the development of lung cancer by antagonizing the effect of *p21*.

## Discussion

Lung cancer is a disease which consists of uncontrolled cell growth in tissues of the lung that may lead to metastases. These growths may ultimately contribute to the majority of the lung cancer deaths. However, the molecular mechanisms linking the initiation and development of lung cancer are not completely understood.

*CDK4 *has gained prominence as a significant cancer-related gene, as its function is to drive cell-cycle progression by phosphorylating the retinoblastoma protein. Overexpression of *CDK4 *has been described in many tumors, including lung cancer.

In this investigation, we analyzed the expression of *CDK4 *protein in lung cancer and normal lung tissues by immunohistochemistry. We found that *CDK4 *was mainly coexpressed in nucleus and cytoplasm in lung cancer tissues and predominantly expressed in cytoplasm in normal lung tissues. Furthermore, we presented evidence that *CDK4 *in nucleus and total protein levels was overexpressed in lung cancer tissues compared to normal lung tissues. Our reports were analogous to Wikman[5], Dobashi[6], and Lingfei[10]*et al*'s results, suggesting that *CDK4 *participates in the pathogenesis of lung cancer.

*CDK4 *is a protein kinase of the *CDK *family that is important for cell cycle G1 phase progression, and its expression pattern is associated with clinical pathology parameters of lung cancer patients. Yoshida *et al. *found that *CDK *was predominantly expressed in low-grade osteosarcomas compared to benign histological mimics, which suggested that *CDK*4 can be a marker distinguishing low-grade osteosarcoma from benign mimics[11]. Zhang *et al. *reported that overexpression of *CDK4 *was positively correlated with Duke's stage of colorectal cancer[12]. In our study, we found that *CDK4 *overexpression was significantly correlated with the status of pathology classification, lymph node metastasis, and clinical stage of lung cancer patients. *CDK4 *appears to be more highly expressed in adenocarcinomas compared to the other two histologic subtypes. Similar to the report from Dobashi *et al.*, we found that overexpression of *CDK4 *was correlated with lymph node metastasis and statistically higher in the N2/N3 group compared to the N0/N1 group[6]. In addition, overexpression of *CDK4 *was positively related to advanced disease status of lung cancer patients. Our results suggested *CDK4 *overexpression in lung cancer may accelerate tumor progression by promoting cell growth.

Further, we presented the evidence that *CDK4 *protein expression in lung cancer was inversely correlated with patient's overall survival. Patients with higher expression of *CDK4 *protein had an overall shorter survival time. According to univariate analysis, patient's overall survival is also inversely proportional to smoking, tumor differentiated degree, and T/N/M classification. Multivariate analyses showed that increased expression of *CDK4 *protein was a significant predictor of poor prognosis for lung cancer patients. Our reports were not consistent with Dobashi [6] and Ghazizadeh's results[13]. The discrepancy is most likely due to the different sample source, sample number, and evaluation method used. However, our results suggest *CDK4 *is a clinical significant biomarker for NPC prognosis.

In previous studies, overexpression of *CDK4 *had been shown to promote cell proliferation by driving cell cycle progression[14-16]. To understand the biological functions of *CDK4 *in lung cancer, we employed a loss-of-function approach by knocking down the expression level of endogenous *CDK4*. To that end, we chose to use lung cancer A549 cell line which express high levels of endogenous *CDK4 *for our study. Similar to results published by Retzer-Lidl, An, and Rodriguez-Puebla *et al. *[14-16], we found that *CDK4 *plays a role in promoting cell proliferation and migration *in vitro*. Furthermore, we also found that inhibition of *CDK4 *could significantly retard the cell cycle transition from G1 to S phase. These results strongly support an oncogenic role for *CDK4 *in the development of lung cancer.

Based on the increased population of G1-S arrested cells after inhibiting *CDK4 *expression, we examined mRNA expression levels of relevant cell cycle factors. *CDK1, CDK2, CDK6, CCND1, p15, p16, p21*, and *p27*[17-21] were first examined in shRNA-*CDK4 *and control cells by real-time PCR. The results indicated that the reduction of endogenous *CDK4 *expression markedly elevated the expression level of tumor suppressor *p21*(≥2 folds). Further, we confirmed the upregulated protein expression of *p21 *in *CDK4*-inhibited cells.

In summary, our results provide evidence that *CDK4 *may be involved in the development of lung cancer. Furthermore, we also demonstrated that *CDK4 *could serve as a potential independent prognostic factor for lung cancer patients. Due to the limited sample size of patients in our investigation, further studies would be needed to verify these findings and establish the role of *CDK4 *as a reliable clinical predictor for lung cancer outcome. Finally, our work is the first to present that *CDK4 *mediates cell cycle progression by regulating the expression of *p21 *expression in lung cancer.

## Competing interests

The authors declare that they have no competing interests.

## Authors' contributions

AW, DW, JG, WL, HY, YZ, XL, HW, and YZ performed this research. WF, ZL and ZY collected, analyzed, and interpreted data and wrote the manuscript. WF, ZL, and ZY supervised all the work. All authors have read and approved the final manuscript.
